# Decision Support Systems in Oncology

**DOI:** 10.1200/CCI.18.00001

**Published:** 2019-02-07

**Authors:** Seán Walsh, Evelyn E.C. de Jong, Janna E. van Timmeren, Abdalla Ibrahim, Inge Compter, Jurgen Peerlings, Sebastian Sanduleanu, Turkey Refaee, Simon Keek, Ruben T.H.M. Larue, Yvonka van Wijk, Aniek J.G. Even, Arthur Jochems, Mohamed S. Barakat, Ralph T.H. Leijenaar, Philippe Lambin

**Affiliations:** ^1^ Maastricht University, Maastricht, the Netherlands

## Abstract

Precision medicine is the future of health care: please watch the animation at https://vimeo.com/241154708. As a technology-intensive and -dependent medical discipline, oncology will be at the vanguard of this impending change. However, to bring about precision medicine, a fundamental conundrum must be solved: Human cognitive capacity, typically constrained to five variables for decision making in the context of the increasing number of available biomarkers and therapeutic options, is a limiting factor to the realization of precision medicine. Given this level of complexity and the restriction of human decision making, current methods are untenable. A solution to this challenge is multifactorial decision support systems (DSSs), continuously learning artificial intelligence platforms that integrate all available data—clinical, imaging, biologic, genetic, cost—to produce validated predictive models. DSSs compare the personalized probable outcomes—toxicity, tumor control, quality of life, cost effectiveness—of various care pathway decisions to ensure optimal efficacy and economy. DSSs can be integrated into the workflows both strategically (at the multidisciplinary tumor board level to support treatment choice, eg, surgery or radiotherapy) and tactically (at the specialist level to support treatment technique, eg, prostate spacer or not). In some countries, the reimbursement of certain treatments, such as proton therapy, is already conditional on the basis that a DSS is used. DSSs have many stakeholders—clinicians, medical directors, medical insurers, patient advocacy groups—and are a natural consequence of big data in health care. Here, we provide an overview of DSSs, their challenges, opportunities, and capacity to improve clinical decision making, with an emphasis on the utility in oncology.

## INTRODUCTION

Decision support systems (DSSs; assistive technology for clinicians, who have limited time and are facing ever-increasing complexity) are hailed as a possible solution to the onerous cognitive burden currently placed on clinicians. However, the potential of DSSs is constrained by rapid-learning health care (RLHC; technology for researchers to collect data across health care networks to facilitate learning and generate knowledge) and artificial intelligence (AI; a computational process to distil actionable insight from data). In simple terms, RLHC can be considered a data mine—an infrastructure from which raw material is obtained for use. AI can be considered a data mill—an apparatus in which raw material is refined for purpose. DSSs are one of the greatest potential benefits of a digital health care ecosystem. Nevertheless, clinically relevant DSSs have been limited in utility and implementation.^1^ This article describes the challenge, the opportunity, and the capacity of DSSs to advance clinical decision making, with a focus on oncology.

## THE CHALLENGE

### Human Cognitive Capacity and Increasing Complexity

**FIG 1.**
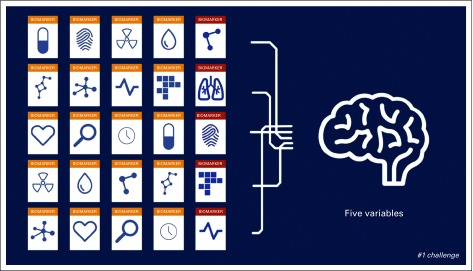
Capacity versus complexity. The upsurge in data available for medical decision making threatens to overwhelm human cognitive capacity (maximum five variables per decision).

The primary challenge, as a consequence of the recent data deluge, is the threat of cognitive overload^1^; A glut of raw data, rather than refined information, confounds the distillation of knowledge and obfuscates decision making (Fig 1).^2^ A study to investigate the limits of human cognitive capacity probed the conceptual complexity of decision making by requesting participants to interpret graphically displayed statistical interactions. In such decisions, all independent variables had be considered together so that decomposition into smaller subtasks was constrained; thus, the order of the interaction directly determined conceptual complexity. As the order of the interaction increased, the number of variables increased. Results showed a large decline in accuracy and speed of solution from three-way to four-way interactions. Furthermore, performance on a five-way interaction was at the chance level.^3^ These findings suggest that a decision based on five variables is the limit of human cognitive capacity. However, the human ability to synthesize information by memory recall/experience to inform intuition is nontrivial for machines to replicate/learn through data capture and should not be overlooked. Nevertheless, this limit must be regarded in the context of precision medicine^4^ (the right treatment, for the right patient, at the right time), a bold new research effort to revolutionize how we improve health and treat disease.^5^ Precision medicine relies on validated biomarkers^6^ (a characteristic that is measured as an indicator of normal biologic processes, pathogenic processes, or responses to an exposure or intervention, including therapeutic interventions^7^) that are integral to the routine management of disease in patients and are used extensively in cancer research and drug development.^8^ Anticancer agents are increasingly being combined with a biomarker to determine which patients are the most likely to benefit from the therapy.^9^ This increase in complexity, coupled with the limits of human cognitive capacity, poses a major challenge for the oncology community.

## THE OPPORTUNITY

### Rapid-Learning Health Care

The threat from data deluge is simultaneously a huge opportunity, because a data-driven RLHC ecosystem will progressively distill and deliver appropriate knowledge to appropriate users within the workflow process, which provides a validated DSS. RLHC is the (re)use of health care data from routine clinical practice and/or clinical trials to support decision making with respect to health care delivery and research.^10^ Issues in RLHC include data representation, standardized nomenclature, data formats and standards, federated data access, data mining and evidence synthesis approaches, evidence retrieval, reporting, and feedback on use of evidence.^11^ Solutions to all of these issues exist and have been implemented in many industries (eg, aviation, automotive, financial) to create global networks and introduce the concept of the Internet of things.^12^ The key to transformation of health care is strategic coordination and facilitation of interoperable approaches to fully realize the innate potential of RLHC.^13^ We must embrace this vision or risk collectively drowning in fragmented data lakes.

### The Cycle

RLHC constitutes four consecutive, infinitely repeated steps^11^ that continuously develop and validate models for DSSs in health care.^14^ The first step is data, which tackles the mining of data (ie, the extraction, transformation, and loading of data, eg, clinical, imaging, biologic, genetic, costs). Procuring data of adequate quality is the greatest opportunity in RLHC. The health care ecosystem must establish a patient-centric, data-driven, knowledge-sharing philosophy across institutional and national borders to benefit from this opportunity. The next step is knowledge, which uses artificial intelligence to distil knowledge from the data (ie, extraction of actionable insight). With AI, machine-learning algorithms analyze data and yield knowledge that can support decisions about new unseen data. Algorithms trained, tuned, and tested on retrospective/prospective data can be used to predict the outcomes (eg, survival, quality of life, toxicity) of various treatments on the basis of data from a new unseen patient. The next step is application, which leverages this knowledge to enhance decision making. The data collected are distilled into knowledge and applied in holistic multifactorial DSSs, intended to support clinicians and patients as they decide the most appropriate course of action (DSSs are neither intended nor suited as a replacement to clinicians in the wider health care context). DSSs must be seamlessly integrated into the clinical workflow to improve efficiency, diminish mistakes, and deliver objectives. The last step of the cycle is evaluation, which measures DSS performance (ie, the sensitivity and specificity of prediction for toxicity, tumor control, quality of life, cost effectiveness). The cycle is repeated perpetually. The essence of the RLHC cycle is that the application of knowledge distilled from data provides deep insight and therefore certainty of decision consequences, which suggests that outcomes can be improved both in terms of effectiveness (realization of the desired result) and efficiency (the resources required to realize the result). Continuous evaluation of RLHC is vital, and the importance of this cannot be overstated. Evaluation should focus on metrics for the questions, “Is the outcome of the treatment as predicted, and, if so, how does this compare with consensus evidence-based guideline knowledge?” Evaluation should be conducted with (a meta-analysis of) robust high-quality data and should be independently interpreted by relevant stakeholders.

### The Five Vs of Big Data

From a scientific perspective, the four Vs (veracity, velocity, variety, and volume)^15^ of big data must to be optimized to fully realize RLHC. The veracity of data is essential to the level of certainty that can be attributed to the knowledge distilled, whereas the velocity of data determines how rapidly and continuously knowledge distillation occurs. Variety of data (in terms of information, not format eg, computed tomography/positron emission tomography/magnetic resonance DICOM imaging [Digital Imaging and Communications in Medicine; the international standard to transmit, store, retrieve, print, process, and display medical imaging information]) enables support of decision making (eg, if all patients are treated radically, you cannot know which patients are overtreated). The volume of data is influential in terms of power (ie, the quality of knowledge distilled from investigations is correlated with the number of patients from whom data were obtained), comprehensiveness (ie, a larger data volume permits the use of more variables in the knowledge step), and exhaustiveness (ie, knowledge related to patients with rare diseases intrinsically requires voluminous data).

From an economic perspective, the fifth V (value) of big data must also be considered. That is, if you are going to invest in the infrastructure required to collect and interpret on a system-wide scale, it is important to ensure that the generated insights are based on accurate data and lead to measurable improvements.

### The Data Disconnect

**FIG 2.**
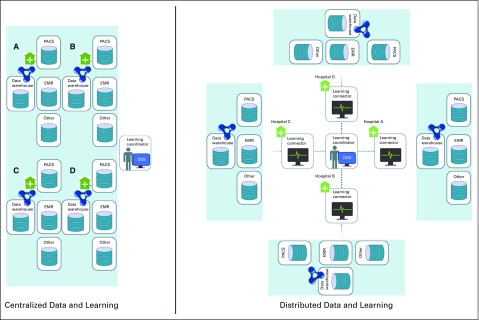
Schematic diagrams of centralized and distributed approaches. In a centralized data and learning approach, multiple centers pool their data to enable learning, whereas, in a distributed data and learning approach, multiple centers link their systems to enable learning. A key aspect of a distributed approach is that it is privacy-by-design construction (ie, data remains at the source), whereas a key aspect of the centralized approach is that data can be directly accessed and scrutinized. These are the two competing tradeoffs between the approaches. DSS, decision support system; EMR, electronic medical record; PACS, picture archiving and communication system.

For RLHC to succeed, data of suitable quality with respect to the five Vs must be procured. Therefore, a motivation exists toward embrace of a data-connected future.^16,17^ However, in the clinical domain, there are several established impediments: inadequate human resources or time, cultural and linguistic difficulties, dissimilarities in data-recording/management methods, the academic/political worth of data, hazards to reputation, legal/privacy deliberations, and more. These impediments, although difficult to overcome, are demonstrably solvable. Two outstanding initiatives to realize the goal of RLHC are CancerLinQ (a centralized data approach^18^) and worldCAT (a distributed data approach^19^; Fig 2). Common efficient solutions via innovative information communication technologies, such as the creation of semantically interoperable data,^20^ which harmonizes local terms to concepts of well-defined ontologies,^21^ are fundamental to the sustained realization of RLHC. Ontology terms act as a collective reference for all data sources, allow a unified process for knowledge distillation from semantically interoperable data, and encourage standardized data management (eg, disease-specific umbrella protocols).^22^

### AI

**FIG 3.**
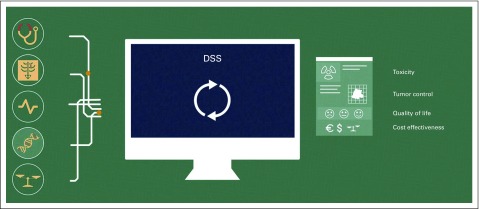
Data-to-decision. Data sources in oncology (clinical, imaging, biologic, genetic, and costs) can be used via artificial intelligence (AI) methods in decision support systems (DSSs) to augment decision making in oncology (toxicity, tumor control, quality of life, cost effectiveness). This volume and complexity of data overload human cognitive capacity but can be mined and distilled by AI in rapid-learning health care frameworks.

AI—the mimicking of human cognition by computers—is a reality in medicine.^23^ AI is an amalgamation of mathematics, computer science, and engineering that implements novel concepts to resolve complex challenges. Machine learning is a subset of AI and has found numerous applications in health care because of the ever-increasing rise in health care complexity.^24^ Recently, deep learning^25^ (in turn a subset of machine learning) has substantially enhanced state-of-the-art speech recognition, language translation, visual object detection, and many other domains, including genomics and drug discovery.^26^ Deep learning discovers complex relationships in data sets through the back-propagation algorithm to guide how a deep neural network (a machine learning model) ought to update its internal parameters that are activated to compute the representation in each layer from the representation in the previous layer. There is a growing consensus that AI (machine learning and deep learning) will be involved more and more in clinical decision making. Therefore, broad implementation of AI algorithms in health care could lead to clinically actionable insight and revolutionize how patients are classified, treatments are developed, diseases are studied, and decisions are made. In oncology, five data sources and four outcomes are typically of interest (Fig 3). To hasten the maturity of AI, clinical and research communities must cultivate an interdisciplinary shared vision of precision medicine. Data must be acquired, curated, standardized, linked, and stored in interoperable and interrogatable databases to realize the extraordinary potential for RLHC that routine standard-of-care data represent.

## THE CAPACITY

### Strategic and Tactical Implementation

**FIG 4.**
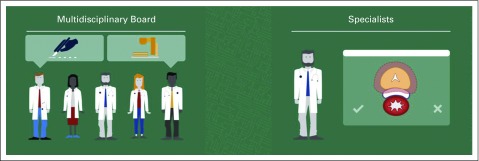
Decision support systems integrated into the workflow. This can be accomplished both strategically (at the multidisciplinary tumor board level to support treatment choice, eg, surgery or radiotherapy) and tactically (at the specialist level to support treatment technique, eg, prostate spacer or not).

DSSs can be built into the workflow strategically (multidisciplinary tumor board level to support treatment choice, eg, surgery or radiotherapy) and tactically (specialist level to support treatment technique, eg, prostate spacer or not; Fig 4). Some nations already condition reimbursement (eg, proton therapy in the Netherlands) on the use of DSSs.

### Stakeholders

**FIG 5.**
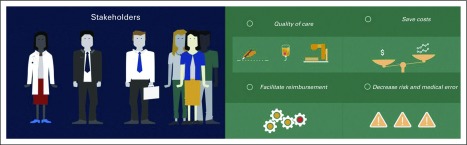
Decision support system (DSS) stakeholders. Clinicians, medical directors, medical insurers, and patient advocacy groups all share common interest in the adoption, use, evaluation, and improvement of DSSs in health care.

The integration of RLHC DSSs into the workflow must be continuously (re-)evaluated by all stakeholders (Fig 5). This evaluation should be performed with (a meta-analysis of) robust data that are independently interpreted by each of the stakeholders and combined into a consensus statement. The guiding light for the stakeholders should be the question, “Is the outcome of treatment as expected, and, if so, how does this relate to consensus and/or evidence-based guideline knowledge?”

### Acceptance and Agency

For DSSs to be widely accepted, frameworks must be created that garner trust from stakeholders.^27^ An important factor for adoption of technologies is ensuring that stakeholders are empowered (ie, the agency to inform, adjust, or reject the DSS) and that their concerns are addressed (eg, for clinicians and patient advocacy groups, increased quality of care and decreased medical errors; for medical directors and insurers, reduced costs and facilitated reimbursement).

### Perception and Provenance

The perception (understanding and inclination) of DSSs by stakeholders is important. Stakeholders should easily comprehend the DSS and desire to use it. Typical heuristics collected from previous implementations of AI into workflows from other industries can be used to develop a nuanced understanding of how stakeholders interact with DSSs to refine interaction patterns and data visualization techniques that work with stakeholders rather than replacing or obstructing them.^27^ In addition, the origin of information immensely influences perception. Stakeholders must have sufficient transparency.

### Shared Decision Making

Health care is shifting toward a more participative, patient-centered approach—an interactive process in which stakeholders collaborate in the selection of health care according to the best available evidence.^10^ DSSs can help patients and clinicians communicate more effectively by providing information and a platform to encourage substantial interaction. DSSs can help patients recognize and clarify their personal values without promotion of one choice over another. This will genuinely deliver personalized and participative therapy that supports both clinicians and patients.

### Translational Potential of DSSs

In the past 5 years (as a result of advances in hardware and software), DSS research has advanced dramatically, which has revealed the potential of this approach to substantially improve clinical care. The information presented in Table 1 provides a nonexhaustive overview of the literature.

**TABLE 1.**
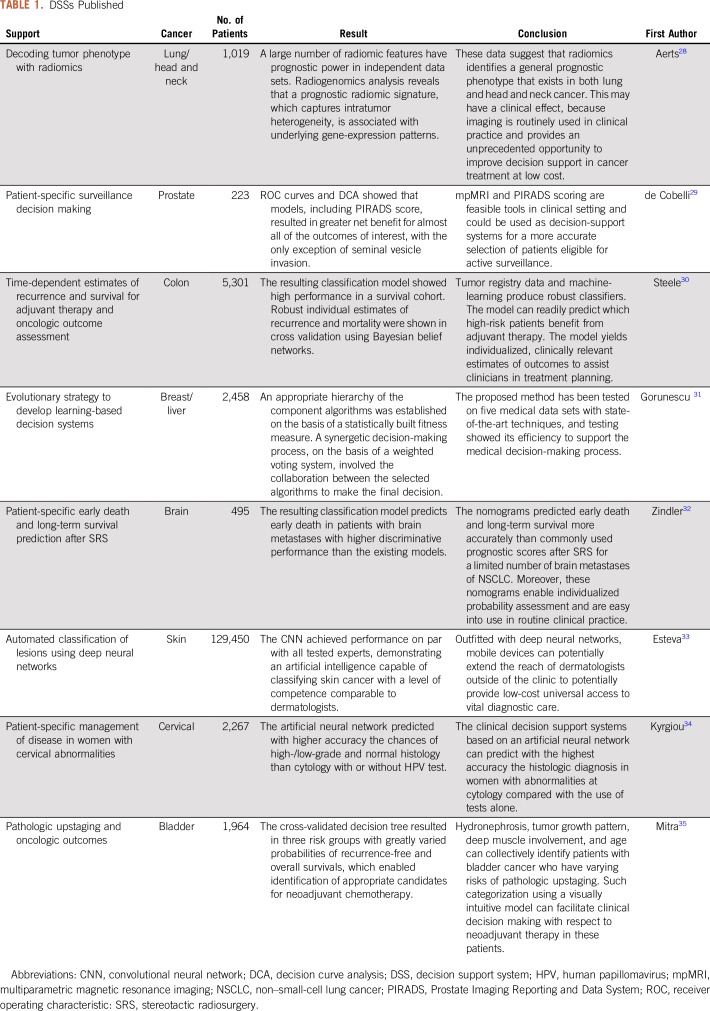
DSSs Published

## DISCUSSION

Human intelligence is vastly superior to AI in general terms (contextualization, association, and reasoning). AI has yet to mature, so DSSs foreseeably will be appropriate for specific tasks only. The role of clinicians will adapt (similar to pilots) as they ally with DSSs, provide expert knowledge, annotate data, and manage performance/efficacy. The users of DSSs must comprehend the benefits and risks. AI can be powerful (ie, automatic detection, localization, classification, interpretation, recommendation, reporting) but also fallible (ie, support of improper decisions caused by presentation of data beyond the training/tuning/testing). Consider the following example: A DSS performs flawlessly after deployment. The department later upgrades hardware and software; what safeguards exist to ensure that the AI does not subsequently produce erroneous assistance, and who is responsible for this?^36^ Another issue is the absence of human intuition about how specific decisions are determined by AI, which leads to unease among many with some declarations that AI is a black box. (However, tools like TensorBoard for TensorFlow^37^ exist to provide transparency.) This deficiency of comprehension hinders adoption by various stakeholders concerned with the ethical/responsible clinical utility of DSSs. To mitigate this, clinicians must actively engage with researchers (academic and industrial) to ensure that the solutions developed yield maximum clinical benefit. Residency programs must adopt AI into curriculums. Clinicians and researchers must work with policymakers on the complexities of DSSs and the consequences of errors (clinical and legal). From a regulatory perspective, despite the perplexity, approval of DSSs by the US Food and Drug Administration and notified bodies within the European Union is happening, notwithstanding the ambiguous working mechanisms. Precedent and parallels to this approach are found in pharmacology: many safe and effective approved drugs have unknown mechanisms of action.^38^

The limit of human cognitive capacity constrains the realization of precision medicine. However, the combination of RLHC and AI to produce DSSs represents a profound opportunity to make precision medicine a reality. DSSs will form part of the future infrastructure and workflow of oncology and will compare the personalized probable outcomes—toxicity, tumor control, quality of life, cost effectiveness—of various care pathway decisions to ensure optimal efficacy and economy. DSSs will strategically and tactically aid all stakeholders.

## Data Availability

The following represents disclosure information provided by authors of this manuscript. All relationships are considered compensated. Relationships are self-held unless noted. I = Immediate Family Member, Inst = My Institution. Relationships may not relate to the subject matter of this manuscript. For more information about ASCO's conflict of interest policy, please refer to www.asco.org/rwc or ascopubs.org/jco/site/ifc. **Employment:** Oncoradiomics **Leadership:** Oncoradiomics **Stock and Other Ownership Interests:** Oncoradiomics **Research Funding: **Varian Medical Systems **Employment:** Medtronic **Employment:** PtTheragnostics **Stock and Other Ownership Interests:** Oncoradiomics **Employment:** PtTheragnostic **Leadership:** PtTheragnostic **Employment:** Oncoradiomics SA **Leadership:** Oncoradiomics SA **Stock and Other Ownership Interests:** Oncoradiomics SA **Patents, Royalties, Other Intellectual Property:** Image analysis method supporting illness development prediction for a neoplasm in a human or animal body (PCT/NL2014/050728) No other potential conflicts of interest were reported.
